# Pesticide use negatively affects bumble bees across European landscapes

**DOI:** 10.1038/s41586-023-06773-3

**Published:** 2023-11-29

**Authors:** Charlie C. Nicholson, Jessica Knapp, Tomasz Kiljanek, Matthias Albrecht, Marie-Pierre Chauzat, Cecilia Costa, Pilar De la Rúa, Alexandra-Maria Klein, Marika Mänd, Simon G. Potts, Oliver Schweiger, Irene Bottero, Elena Cini, Joachim R. de Miranda, Gennaro Di Prisco, Christophe Dominik, Simon Hodge, Vera Kaunath, Anina Knauer, Marion Laurent, Vicente Martínez-López, Piotr Medrzycki, Maria Helena Pereira-Peixoto, Risto Raimets, Janine M. Schwarz, Deepa Senapathi, Giovanni Tamburini, Mark J. F. Brown, Jane C. Stout, Maj Rundlöf

**Affiliations:** 1 Department of Biology, Lund University, Lund, Sweden; 2 School of Natural Sciences, Trinity College Dublin, Dublin, Ireland; 3 Department of Pharmacology and Toxicology, National Veterinary Research Institute, Puławy, Poland; 4 Agroscope, Agroecology and Environment, Zurich, Switzerland; 5 Laboratory for Animal Health, ANSES, Paris-Est University, Maisons-Alfort, France; 6 Council for Agricultural Research and Economics—Agriculture and Environment Research Centre, Bologna, Italy; 7 Department of Zoology and Physical Anthropology, University of Murcia, Murcia, Spain; 8 Nature Conservation and Landscape Ecology, University of Freiburg, Freiburg, Germany; 9 Institute of Agricultural and Environmental Sciences, Estonian University of Life Sciences, Tartu, Estonia; 10 Centre for Agri-Environmental Research, School of Agriculture, Policy and Development, University of Reading, Reading, UK; 11 Department of Community Ecology, Helmholtz Centre for Environmental Research—UFZ, Halle, Germany; 12 German Centre for Integrative Biodiversity Research (iDiv) Halle-Jena-Leipzig, Leipzig, Germany; 13 Department of Ecology, Swedish University of Agricultural Sciences, Uppsala, Sweden; 14 Institute for Sustainable Plant Protection, The Italian National Research Council, Portici, Italy; 15 Unit of Honey Bee Pathology, Sophia Antipolis Laboratory, ANSES, Sophia Antipolis, France; 16 Department of Soil, Plant and Food Sciences, University of Bari, Bari, Italy; 17 Department of Biological Sciences, Royal Holloway University of London, Egham, UK

**Keywords:** Environmental impact, Agroecology, Conservation biology, Sustainability, Environmental monitoring

## Abstract

Sustainable agriculture requires balancing crop yields with the effects of pesticides on non-target organisms, such as bees and other crop pollinators. Field studies demonstrated that agricultural use of neonicotinoid insecticides can negatively affect wild bee species^1,2^, leading to restrictions on these compounds^3^. However, besides neonicotinoids, field-based evidence of the effects of landscape pesticide exposure on wild bees is lacking. Bees encounter many pesticides in agricultural landscapes^4–9^ and the effects of this landscape exposure on colony growth and development of any bee species remains unknown. Here we show that the many pesticides found in bumble bee-collected pollen are associated with reduced colony performance during crop bloom, especially in simplified landscapes with intensive agricultural practices. Our results from 316 *Bombus terrestris* colonies at 106 agricultural sites across eight European countries confirm that the regulatory system fails to sufficiently prevent pesticide-related impacts on non-target organisms, even for a eusocial pollinator species in which colony size may buffer against such impacts^10,11^. These findings support the need for postapproval monitoring of both pesticide exposure and effects to confirm that the regulatory process is sufficiently protective in limiting the collateral environmental damage of agricultural pesticide use.

## Abstract (web summary)

Results from 316 *Bombus terrestris* colonies at 106 agricultural sites across eight European countries find pesticides in bumble bee pollen to be associated with reduced colony performance, especially in areas of intensive agriculture.

## Main

Reliance on chemical pest control has created contaminated agricultural landscapes that expose bees to many pesticides^4–9,12^. Agricultural uses of neonicotinoid insecticides have been in the spotlight for their negative effects on bees^1,2,13,14^ but it is unknown how effects scale beyond single substances in focal fields. We still do not know the consequences of landscape-level pesticide exposure, which results from agricultural uses of multiple approved pesticides over pollinator-relevant spatiotemporal scales, on the growth and development of any bee species. Here we empirically test the effects of landscape pesticide exposure on the key wild and commercial bumble bee pollinator *Bombus terrestris* L., answering recent calls for realistic pesticide mixture risk assessment at landscape scales^15^.

**Fig. 1:**
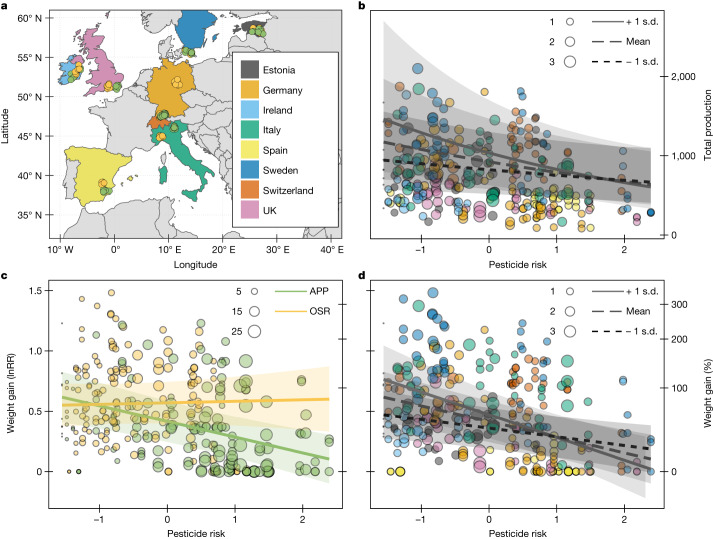
Effects of landscape exposure to pesticides on bumble bee colony weight and production. **a**, We deployed bumble bee (*B. terrestris* L.) colonies (*n* = 316) adjacent to apple (APP, green points) and oilseed rape (OSR, yellow points) across eight European countries. **b**, Colony production (total number of produced bees estimated by the sum of closed and eclosed cocoons) declined with pesticide risk (log-transformed and centred toxicity-weighted pesticide concentrations in pollen stores; Methods). **c**,**d**, Colony weight gain (response ratio ln(*g*_max_/*g*_initial_) and percentage change (exp(lnRR)) also declined with pesticide risk (note double and shared *y* axes). Focal crop (**c**) and landscape context (**b**,**d**) modified these effects, with stronger declines at apple (**c**; green line) compared to at oilseed rape sites (**c**; yellow line) and in landscapes with more cropland (**b**,**d**; solid line +1 s.d. proportion of cropland). Point colours (**b**,**d**) correspond to country colours (**a**) and are scaled by their MCR, the factor by which the mixture of compounds in a sample is riskier than the single most risky compound (Methods). Points in **c** are scaled by the number of pesticide compounds quantified in a sample. Fitted lines are estimated on the basis of generalized (**b**) and linear (**c**,**d**) mixed effects models. Shaded areas represent the regression 95% CI. Results from statistical models are given in Table 1.

As central place foragers, the fitness of bees depends on the net value of forage resources in their foraging range, which can be reduced if these resources are contaminated with hazardous pesticides^7,8,16^. Thus, intensively managed agricultural landscapes, with fewer flowers and seminatural habitats and simplified cropping systems with increased reliance on pesticides, are likely to increase the risk of pesticide exposure to bees^8,17,18^. Likewise, crops with different pesticide-use regimes and attractiveness to pollinators will also influence the exposure and risk of pesticides for bees^7,19^. To empirically test the consequences of landscape pesticide exposure, we placed sentinel colonies of *B. terrestris* (*n* = 316) along a gradient of the proportion cropland in the surrounding landscape (range 3–98%) at agricultural sites growing two focal flowering crops (apple *n* = 50 and oilseed rape *n* = 56) across eight European countries (Fig. 1a). We collected pollen samples from the colonies, which were screened for 267 compounds (Supplementary Table 1) to quantify pesticide residues.

**Extended Data Fig. 1:**
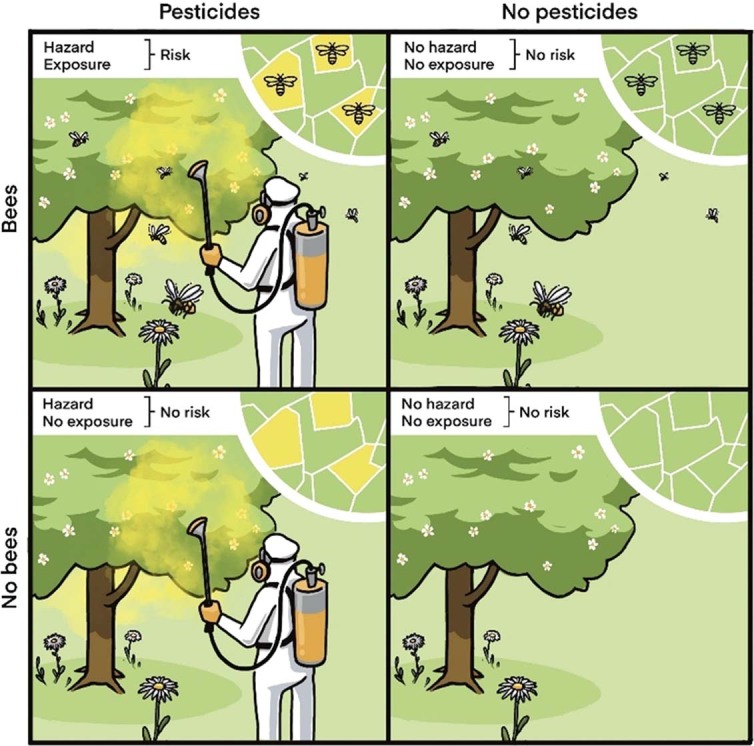
A simplified view of landscape exposure and resulting pesticide risk to bees. Pesticide use creates potential hazard for non-target organisms. For bees in agricultural landscapes, pesticide risk results when their activity exposes them to this hazard (top left panel). Without the co-occurrence of hazard and exposure we expect no risk (remaining panels). Of course, the degree of hazard and exposure will depend on pesticide properties (e.g., toxicity, environmental fate, product formulations, use patterns) and bee traits (e.g., foraging range, sociality, body size, detoxification pathways). Moreover, real-world exposure occurs at landscape scales (see insets), because bees can integrate multiple sources of exposure by visiting spatially separated patches that vary in the identity, amount, timing and toxicity of hazard. We use the colony pollen stores collected by bumble bees (*Bombus terrestris*) to quantify pesticide risk resulting from this landscape exposure. We quantify exposure as the concentrations (µg/kg) of 267 substances in the pollen while hazard is quantified by the substances’ toxicities (LD_50s_). Scaling concentrations by toxicities and summing these toxicity-weighted concentrations provides a relative measure of pesticide risk to bees.

We tracked bumble bee colony performance by weighing colonies before, during and after focal crop bloom and by counting all bees at colony termination after bloom. We relate these colony performance endpoints to pesticide risk (summed toxicity-weighted pesticide concentrations in pollen; Methods) resulting from landscape exposure (Extended Data Fig. 1). We found that increasing pesticide risk reduced bumble bee colony production (summed eclosed and closed cocoons of all castes; Methods) and this effect was modified by an interaction with the proportion of cropland in the surrounding landscape (Fig. 1b and Table 1; generalized linear mixed effects model (GLMM): *χ*^2^ (1, 307) = 5.46, *P* = 0.019). Gain in colony weight—a metric inclusive of bees, brood and food—also decreased with increasing pesticide risk and focal crop (Fig. 1c; linear mixed effect model (LMM): *χ*^2^ (1, 307) = 9.13, *P* = 0.0025), as well as the proportion of cropland in the surrounding landscape (Fig. 1d; LMM: *χ*^2^ (1, 307) = 10.60, *P* = 0.001) modified this effect (Table 1). Colony weight gain was smaller with increasing pesticide risk when apple was the focal crop (slope estimate (95% confidence interval [CI]): −0.13 [−0.19, −0.07]) but not at the more resource-yielding oilseed rape^20^ (0.02 [−0.06, 0.08]; Fig. 1c), suggesting that higher flower resource availability can mitigate the negative effects of pesticides on bees^8,21^. Colony production (Fig. 1b) and weight gain (Fig. 1d) decreased more with increasing pesticide risk in landscapes with a higher proportion of cropland (more than 75%) compared to a lower proportion of cropland (less than 34%). Simplified landscapes, dominated by non-flowering cropland, generally contain fewer flower resources^22,23^, potentially stressing colonies and interacting with pesticide effects^24,25^. Likewise, high pesticide risk may hamper the bees’ foraging efficiency^26^, an already difficult task in resource-poor environments.

**Table 1:** Effects of pesticide risk, crop identity and proportion of cropland on colony production and weight gain

	Production of bees		Weight gain	
	*χ*²	*P*	χ²	*P*
Initial weight	0.22	0.63000	20.86	<0.0001
Risk	4.99	0.02500	10.77	0.0010
Crop	12.26	0.00046	8.10	0.0044
Cropland	1.35	0.25000	1.31	0.2500
Risk × crop	3.98	0.04500	9.14	0.0025
Risk × cropland	5.46	0.01900	10.60	0.0011
Crop × cropland	4.95	0.02600	3.47	0.0620
Risk × crop × cropland	0.79	0.37000	0.60	0.4400
*R*^2^ marginal	0.19		0.23	
*R*^2^ conditional	0.58		0.76	

Colony total production of bees (sum of eclosed and intact cocoons) and weight gain (log response ratio) in relation to initial colony weight, pesticide risk (sum of toxicity-weighted pesticide concentrations; Methods), crop type (oilseed rape and apple), proportion of cropland in the surrounding landscape (1 km radius) and their interactions.

Colony pollen stores contained many pesticides (95% with more than 1 compound; median 8; range 1–27), with more unique compounds in apple (80) than in oilseed rape (68). Although fungicides comprised 81% of total residues (µg kg^−1^) and 62% of compound quantifications, insecticides represented most of the risk, with 99% of risk coming from nine insecticide compounds (Table 2). These high-risk compounds included the known bee health antagonists imidacloprid and indoxacarb, as well as pyrethroids and organophosphates (Table 2). Most pollen samples (62%) have maximum cumulative ratios (MCRs)—the factor by which risk from all compounds was greater than its most risky compound (Methods)—less than 1.5 (range 1–3.8) (Fig. 1b,d). Together, these results indicate that pollen stores often contain many pesticides but that high concentrations of a few highly toxic insecticide compounds determine most of the mixture pesticide risk (Supplementary Table 2).

**Table 2:** Ten compounds found in the colony pollen stores posing most risk to bumble bees in European agricultural landscapes

Pesticide (type)	Chemical group	LD_50_ mean	LOQ	Concentration mean	Concentration median	Concentration 90th percentile	Frequency	Compound risk
Indoxacarb (I)	Oxadiazine	0.1560	5.0	1,310	57	3,380	17 (16%)	1,430
Spinosad (I)	Spinosyn	0.0303	5.0	658	658	1,170	2 (2%)	434
Chlorpyrifos-Ethyl (I)	Organophosphate	0.1090	5.0	282	13.9	561	9 (8%)	233
Deltamethrin (I)	Pyrethroid	0.0358	5.0	68.80	68.8	117	2 (2%)	38.50
Dimethoate (I)	Organophosphate	0.1000	1.0	31	15.4	77.3	11 (10%)	34.10
Imidacloprid (I)	Neonicotinoid	0.0424	1.0	9.490	8.1	17.5	9 (8%)	20.20
Cyfluthrin (I)	Pyrethroid	0.0255	1.0	41.50	41.5	41.5	1 (1%)	16.30
Dithianon (F)	Quinone	62.700^a^	50.0	3,300	244	12,900	25 (24%)	12.60
Etofenprox (I)	Pyrethroid	0.2020	5.0	61.90	47.5	91.9	3 (3%)	9.19
Chlorpyrifos-Methyl (I)	Organophosphate	0.1620	5.0	36.90	16.6	80.9	4 (4%)	9.08

Pesticide identity, type (I, insecticide; F, fungicide), chemical group, toxicity (average acute oral and contact LD_50_ (dose required to cause 50% mortality in the test population) for *A. mellifera* adults, considering worst case from 24, 48 and 72 h values, µg per bee^46^), limit of quantification (LOQ) (μg kg^−1^), concentrations (mean, median, 90th percentile; µg kg^−1^), frequency of quantification (number of sites out of 106 sites with positive samples) and individual compound risk (Methods) of the ten riskiest pesticide compounds in colony pollen stores.

^a^LD_50_ based on limit test.

**Extended Data Fig. 2:**
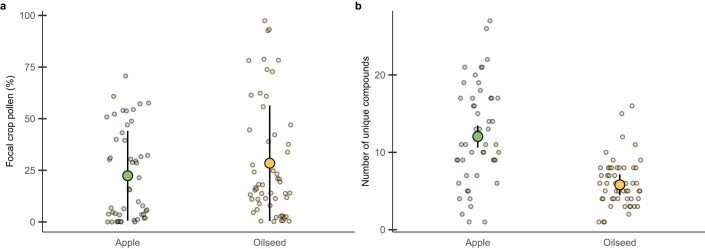
Percent of focal crop pollen and the number of pesticide compounds in pollen stores. Bumble bee colony stores contained a substantial but variable portion of focal crop pollen types (**a**). More pesticide compounds were found in pollen collected at apple (*n* = 50) sites than at oilseed rape (*n* = 56) sites (**b**, *F*_1,104_ = 39.59, *P* < 0.001). For proportion of focal crop pollen (**a**), large points are means based on raw data and error bars are standard deviations. For the number of unique compounds (**b**), large points are estimated means from linear models and error bars are 95% confidence intervals. Small points are the individual data points.

Focal crop pollen contributed a substantial but variable portion of the colony pollen stores (22 ± 22% at apple sites and 28 ± 28% at oilseed rape sites; mean ± s.d*.*; Extended Data Fig. 2a) and was not related to the proportion of cropland in the landscape (*χ*^2^ (1, 103) = 0.25, *P* = 0.62). Colony pollen stores at apple sites contained more pesticide compounds (Fig. 1c and Extended Data Fig. 2b). Apple and other fruit crops generally have higher pesticide use^27^ and thus higher pesticide risk for bees, than do annual arable crops^5,7^ or diversified farmland with permanent grasslands^28,29^. This reliance on many pesticides for pest management may increase the co-occurrence of compounds with known synergies, such as azole fungicides or cholinesterase-inhibiting insecticides^30^. Thus, our risk metric may underestimate or overestimate the potency of pesticide mixtures in agricultural landscapes because it assumes risk additivity of mixtures. Nonetheless, synergism among pesticides is relatively rare^30^ and assuming concentration addition is considered a reasonable starting point in regulatory risk assessment of mixtures^31^.

**Extended Data Fig. 3:**
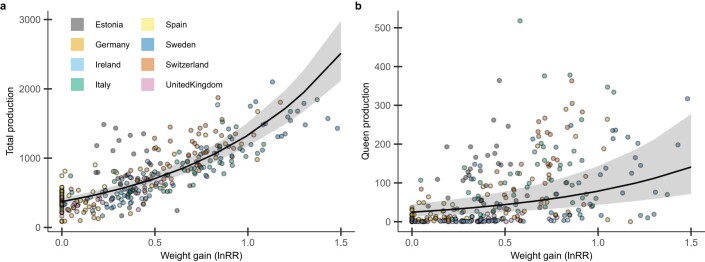
Bumble bee colony performance metrics are correlated. Colony weight gain (response ratio: ln(g_max_/g_initial_)) is positively related with (**a**) total colony production (total number of produced bees estimated by the sum of closed and eclosed cocoons; *χ*² = 354.27, *P* < 0.001) and (**b**) queen production (sum of closed and eclosed queen cocoons; *χ*² = 37.42, *P* < 0.001). Fitted lines are estimated based on generalized linear mixed effects models with a negative binomial error distribution. Shaded areas represent the regression 95% confidence intervals. Point colours correspond to country colours in Fig. 1a and Extended Data Fig. 5a.

**Extended Data Fig. 4:**
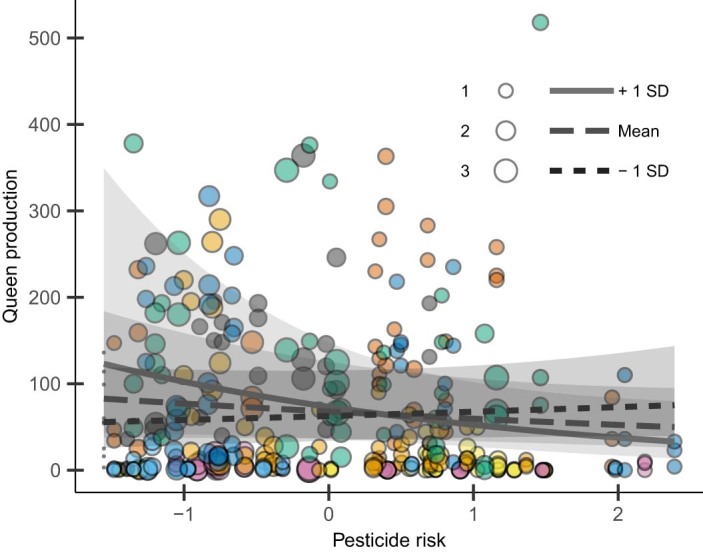
Effects of field exposure to pesticides on bumble bee colony queen production. Colony queen production (sum of closed and eclosed queen cocoons) declined with pesticide risk (centred toxicity-weighted pesticide concentrations in pollen stores, see Methods) and landscape proportion cropland modified this effect, with stronger declines in landscapes with more cropland (solid line +1 *SD* proportion of cropland). Points are scaled by pesticide maximum cumulative ratio (MCR), the factor by which the mixture of compounds is riskier than the single most risky compound (see Methods). Fitted lines are estimated based on generalized linear mixed effects models with a negative binomial error distribution. Shaded areas and error bars represent the regression 95% confidence intervals. Results from statistical models are given in Extended Data Table 1. Point colours correspond to country colours in Fig. 1a and Extended Data Fig. 5a.

**Extended Data Fig. 5:**
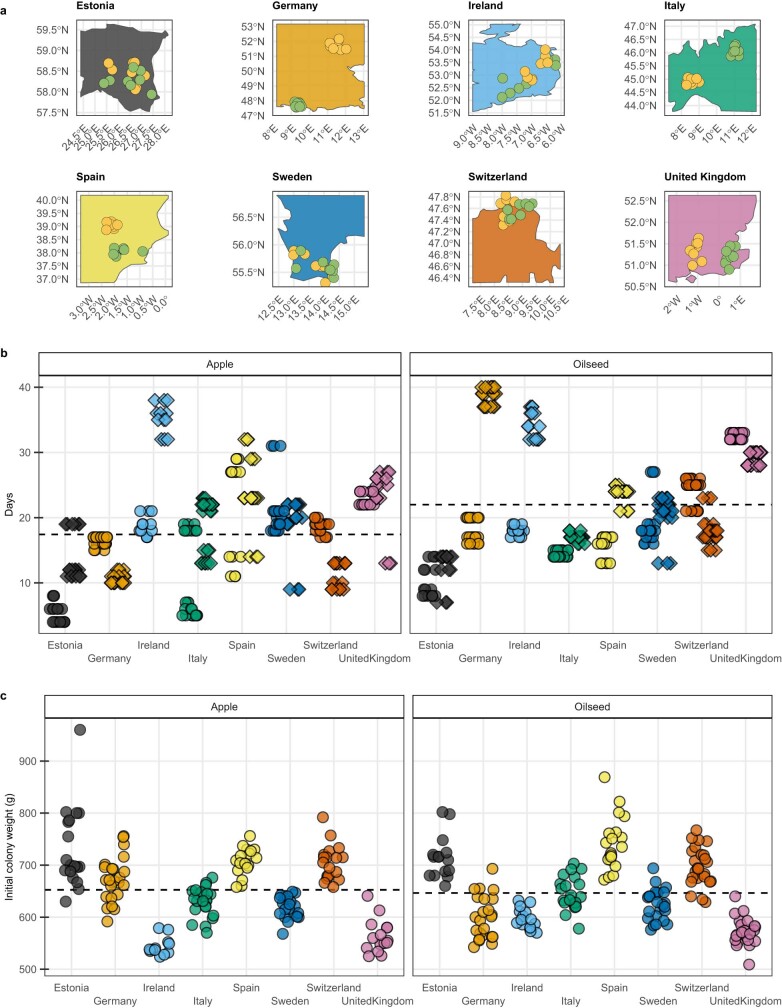
Overview of study design and set-up. Our research network (**a**) included 128 sites in two focal crops (apple: green points, oilseed rape: yellow points) in eight European countries. At each site, three bumble bee (*Bombus terrestris*) colonies were deployed prior to focal crop bloom and weighed three times: before, during and after focal crop bloom. (**b**) The interval between first and second weights (circles) and second and third weights (diamonds) varied depending on region- and crop-specific bloom periods. (**c**) Colony weights at the time of deployment. Crop averages for number of days (**a**) and initial weight (**b**) across colonies are given as dashed lines. Site coordinates (**a**) are randomly jittered to protect farmer confidentiality. Colours in **b** and **c** correspond to country colours in **a** and Fig. 1a.

Mass-flowering crops such as oilseed rape can increase bumble bee colony growth when not accounting for pesticide exposure^32^, especially when flowering coincides with peak worker numbers^33^. Therefore, we specifically timed colony placement to coincide with focal crop bloom, so that colony performance could be influenced by the net value of the focal crop: its nutritional benefit, minus pesticide cost. Bumble bee colony weight gain correlates with total production (Extended Data Fig. 3a), including queens (Extended Data Fig. 3b) and males^34,35^, so our findings suggest the potential for adverse effects of pesticides on reproduction and subsequent population dynamics of bumble bees^36^. Indeed, we see that the production of new queens declined with increasing risk similarly to weight gain (Extended Data Table 1 and Extended Data Fig. 4). However, our approach meant colonies were at sites for different durations (apple 36.3 ± 11.4 days (mean ± s.d.); oilseed rape 43.0 ± 12.2 days; Extended Data Fig. 5) depending on region- and crop-specific bloom periods, precluding examination of full reproductive output and weight dynamics over the complete colony cycle, which follows an exponential growth and decline^37^.

Understanding how and to what extent different cropping patterns and landscape contexts put key pollinator species at risk is essential for accurate and reliable pesticide risk assessment^15,38^. Our findings from 106 landscapes across Europe confirm that agricultural pesticide use results in exposure to many pesticides that reduce bumble bee colony performance during crop bloom, especially in simplified landscapes. Furthermore, our results can guide future postapproval monitoring efforts of non-target effects from landscape pesticide exposure^39^. *Bombus terrestris* is a valuable sentinel of the broader bee community for monitoring pesticide exposure^7^ and effect^1^ because its life history traits, such as colony size and foraging capacity, are intermediate to *Apis mellifera* and most solitary bee species. Nonetheless, *B. terrestris* forms colonies that may buffer the severity of pesticide effects^10,11^. Thus, the effects observed in our study may be more severe for the numerous solitary- and smaller-colony bee species^40,41^.

**Extended Data Fig. 6:**
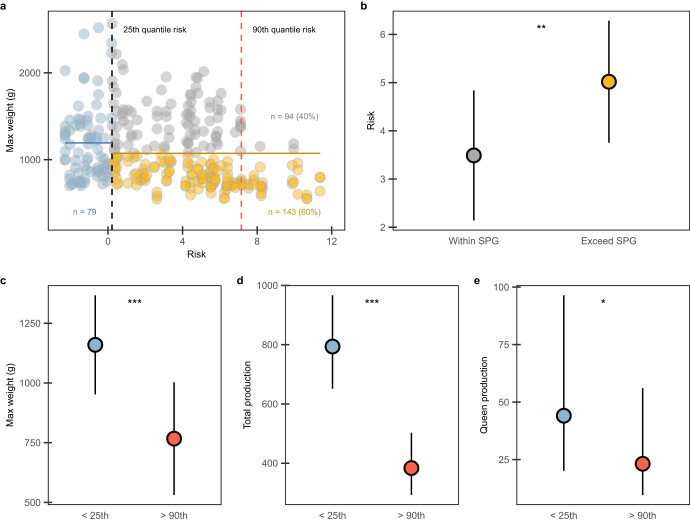
Pesticide risk effects exceed a suggested Specific Protection Goal (SPG). To evaluate the magnitude of pesticide risk for the bees (**a**), we assume that the colonies belonging to a low-risk group (blue points, 25th percentile of risk) can be used to calculate the average maximum weight as a baseline (blue line). Using a suggested SPG for bumble bees of 10% reduction in colony weight^42^ (yellow line), 60% of the remaining colonies in our study exceed this. In (**b**) we compare the risk for colonies that exceed the SPG (yellow points; n = 143 colonies) to those that do not (grey points; n = 94). The SPG is meant to protect 90% of the colony population across Europe, therefore in (**c-e**) we compare colony performance endpoints between the baseline colony group (blue, 25^th^ percentile of risk; n = 79) and a high-risk colony group (red, 90th percentile of risk; n = 30) based on (**a**). Points and error bars (**b–e**) depict estimated means and 95% confidence intervals from linear (**b,c**) and generalized linear mixed effects models (**d,e**). * P < 0.05, ** P < 0.01 and *** P < 0.001. Exact p-values from statistical models are given in Extended Data Table 2.

**Extended Data Fig. 7:**
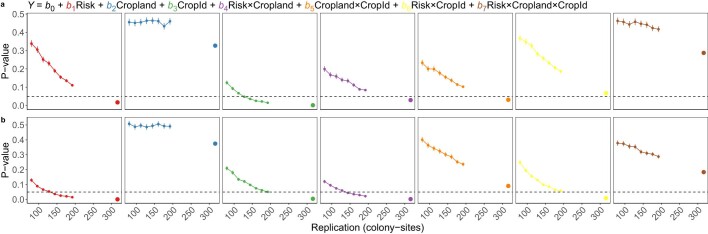
Sensitivity analysis of mixed effect models for the effect of pesticide risk, focal crop and proportion cropland. We specified the same model structure (equation above plots) and simulated different levels of replication through stratified subsampling without replacement of colonies at the focal crop-country level and repeated the analyses for weight gain (**a**) and production (**b**) with these rarefied data sets. Levels of replication spanned 5 colonies per focal crop and country (*N* = 80 colonies) to 12 (*N* = 192), with this later value being the lowest focal crop-country level of replication in the data. The large point depicts the level of replication (*N* = 316 colonies) and p-values reported in the main text (See Table 1). Points and error bars (**a,b**) are means and 95% confidence intervals calculated over 1000 iterations per replication level.

Our results provide robust, European-wide evidence that landscape pesticide exposure negatively affects non-target organisms in agricultural landscapes. Using the average maximum weight of low-risk colonies (that is, the 25th percentile of risk) as a baseline, we found that 60% of remaining colonies exceed a current suggested specific protection goal (SPG) for bumble bees (10% colony weight reduction^42^; Extended Data Fig. 6a) and that these colonies were more at risk (Extended Data Fig. 6b). Further, compared to low-risk colonies, we observed a 34% reduction in maximum weight (estimated mean difference 393 g; Extended Data Fig. 6c), 52% reduction in total production (410 individuals; Extended Data Fig. 6d) and a 47% reduction in queen production (21 individuals; Extended Data Fig. 6e) in the high-risk group (that is, the 90th percentile of risk). Thus, the European pesticide regulatory system for pesticides is not sufficiently protective given this SPG, indicating the need for postapproval monitoring of landscape exposure and its effects^15,24,39^. However, field-based assessments, as we present here, require high amounts of replication^43^ and post hoc sensitivity analysis shows that more than 150 colony–site combinations are required to detect the effects we observed (Extended Data Fig. 7). In silico approaches to predict bee health are promising for a more holistic environmental risk assessment^15^, for which these results could form an empirical basis.

Our results show that ambitious sustainability goals related to pesticide reduction—objectives of the COP 15 meeting on the Convention on Biological Diversity^44^ and the European Farm to Fork strategy^45^—would benefit bee populations and potentially the pollination services they provide^46^. Conversely, the current assumption of pesticide regulation—that chemicals that individually pass laboratory tests and semifield trials are considered environmentally benign—fails to safeguard bees and other pollinators that support agricultural production and wild plant pollination. Thus, future monitoring of bee populations under typical agricultural practices, accounting for landscape exposure, is a vital step towards a system of pollinator pesticidovigilance^39^.

## Methods

### Study landscapes

Our site network spanned 128 agricultural sites in eight European countries encompassing many biogeographic zones with differing climates and seasonality^47^ (Fig. 1a). Sites focused on either oilseed rape or apple crops. In each focal crop, sites were selected to occur along a gradient of proportion of cropland within 1 km radius landscapes. This proportion is an established proxy for the agricultural management intensity typical of each country^47^. We chose oilseed rape and apple as our focal crops to reflect annual and perennial cropping practices and, therefore, different pest pressures, pest management strategies and pesticide use^27,48^. Furthermore, these crops are grown throughout Europe and so provided standardization across this geographic range. Apple and oilseed rape provide abundant food resources for pollinators^49^, require pollination^50^ and are economically important^51^, reiterating the need for reliable ecosystem services in these landscapes. The most dominant land cover types were cropland (mean 55%; range 3–98%) and seminatural areas (mean 37%; range 0.1–93%), where the latter comprised grasslands (mean 19%; range 0.1–76%), woodlands (mean 18%; range 0–62%) and wetlands (mean 0.1%; range 0–3%). These two dominant land covers were strongly negatively correlated (*R*_104_ = −0.95, *P* < 0.001). All sites were more than 3 km apart to ensure the spatial independence of the bumble bee colonies, whose foraging range is generally less than 1.5 km (ref. ^52^).

### Sentinel colonies and measurements of colony performance during crop bloom

**Extended Data Fig. 8:**
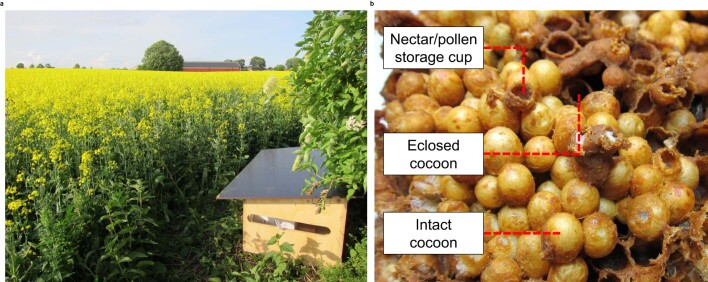
***Bombus terrestris*** colonies in the field (**a**) and under dissection in the laboratory (**b**).

At each site, we used three bumble bee colonies (*B. terrestris terrestris* for continental Europe and *B. terrestris audax* for the UK and Ireland) (*n* = 384), housed in protective structures (Extended Data Fig. 8), before focal crop bloom in 2019. Before deployment, we confirmed that each colony had a natal queen and recorded its initial weight (648 ± 70.9 g (mean ± s.d.); Extended Data Fig. 5c). Colonies were weighed again during peak bloom of the focal crop in each country. At the end of the crop bloom, colonies were weighed again, then sealed, retrieved from sites and frozen. Of the 384 colonies initially deployed across 128 sites (64 apple, 64 oilseed rape), we analysed 316 colonies from 106 sites. This reduced sample size is due to colony losses (for example, animal attack and overrun by machinery; *n* = 5) or colonies not yielding enough stored pollen material for pesticide quantification (*n* = 63; Supplementary Table 3). The last could potentially be avoided in any future studies by complementing with concurrent collection of returning foragers’ corbicular pollen^7,8^.

In the laboratory, we removed any wax covering and sorted through the colony structure to count the number of intact and eclosed worker/male and queen cocoons (Extended Data Fig. 8), on the basis of their different size^1^. Our approach allowed us to derive two main indices of colony performance: (1) colony weight gain and (2) the total colony production. For weight gain, we calculated the natural-log response ratio for each colony as ln(*g*_max_/*g*_initial_), where *g*_initial_ is weight before bloom and *g*_max_ is the maximum weight achieved by a colony during its field placement. In most cases (62% of colonies), *g*_max_ was achieved by the final weighing but, in some cases, *g*_max_ was achieved at the second (26%) or first (12%) weighing. For total colony production, we summed the number of intact and eclosed cocoons, including the eclosed cocoons used for nectar and pollen storage, instead of the number of bee individuals present at the time of colony termination, as new reproductives (gynes and males) could have left the colony at the time of retrieval. In addition, we summed the number of intact and eclosed queen cocoons for an indication of the colony reproduction. Colony termination was timed to crop bloom, rather than colony dynamics, preventing colony cycle completion and full reproduction. Queen production should therefore be interpreted with caution. During colony dissection, we extracted pollen stored in colonies (Extended Data Fig. 8), pooling from all three colonies aiming for at least 15 g but using samples down to 0.52 g for pesticide residue analysis (*n* = 106 pollen samples). Samples were homogenized before preparing subsamples for palynological and pesticide residue analyses. All samples were stored at −20 °C.

### Palynological analysis

Palynological analyses were performed at the Research Centre for Agriculture and Environment (CREA) Bologna, Italy. For each homogenized pollen store sample, 1.0 g was dissolved in 20 ml of distilled water. Using a Pasteur pipette, a drop of sediment was placed on a microscope slide and spread out over an area about 18 × 18 mm^2^. After drying, the sediment was included in glycerine jelly and covered with the cover slip. Examination under the microscope was performed with ×400 magnification. After a first read to identify all the pollen types in the slide, a second read of the slide was carried out until 500 pollen grains were counted. Abortive, irregular or broken pollen grains were counted if they could be identified. Non-identifiable or non-identified grains were noted separately. Recognition of pollen type was based on comparison between the observed pollen forms and those present in the CREA collection of reference slides (a database with more than 1,000 thermophilous species developed using anthers of identified plant species). For each pollen type, the percentage with respect to the total number of counted pollen grains was calculated.

### Pesticide residue analysis

Pesticide residue analyses were performed at the Department of Pharmacology and Toxicology, National Veterinary Research Institute, Puławy, Poland, which is the National Reference Laboratory for pesticide residue analysis and regularly participates in international proficiency tests with satisfactory results. We used 0.3 g of homogenized pollen store samples to screen for 267 compounds including isomers and metabolites (Supplementary Table 1). Particular attention was paid to analysing pesticides that are the active substances in plant protection products recommended for the protection of oilseed rape and apple orchards^53,54^. We use a previously described method^55^ that is validated according to SANTE/12682/2019 (ref. ^56^) and accredited in accordance with the ISO 17025 standard. First, a sample was extracted with 1 ml of a solution of 5% formic acid in acetonitrile, and then the ammonium formate salt was added. The extract was subjected to clean-up by freezing and two-step dispersive solid phase extraction with a Supel QuE Verde sorbents. After first step dispersive solid phase extraction (dSPE), a portion of extract was analysed by liquid chromatography tandem mass spectrometry system (Agilent 1260 HPLC coupled with an AB Sciex QTRAP 6500 mass spectrometer) for 200 pesticide residues. The remaining extract was subjected to second step dSPE clean-up by another Supel QuE Verde and then, after concentration and solvent exchange, was analysed by gas chromatography tandem mass spectrometry (Agilent GC 7890 A+ coupled with a 7000B mass spectrometer) for another 61 pesticide and 6 ndl-PCB residues. Procedural standard calibration was used for calibration^56^. Reagent blanks and blank samples were analysed in each batch. Recovery checks with samples spiked with pesticides at limit of quantification (LOQ) levels were performed in each analytical batch to meet SANTE/12682/2019 criteria^56^.

### Calculation of pesticide risk

We use toxicity-weighted concentrations (TWC) as a basis for indicating the direct pesticide risk to bees^7,8^, where the TWC for each compound (TWC_*i*_) is the ratio of its concentration in bee-collected pollen (µg kg^−1^; *c*_*i*_) and its respective acute toxicity endpoint (LD_50*i*_—the dose required to cause 50% mortality in the test population). Following a concentration addition approach, the recommended default for mixture environmental risk assessment^31,57^, we summed TWCs to calculate the additive toxicity-weighted concentration of all compounds within a sample per site (TWC_mix_):

$${{\rm{TWC}}}_{{\rm{mix}}}=\mathop{\sum }\limits_{i=1}^{n}\frac{{c}_{i}}{{{\rm{LD}}}_{{\rm{50}}}i}$$

We used an average of the acute oral and contact lethal doses LD_50_ for each compound sourced from the Pesticide Properties Database^58,59^ to provide an overall indicator of toxicity, reflective of how bees encounter pesticides in the landscape, that is, moving contaminated food in contact with their bodies for oral consumption^60^. We used the LD_50_ for adult *A. mellifera* because there are incomplete toxicity data for other bee species and, if there are data, intertaxa correlation is high^60,61^. We rounded LD_50_ down when based on limit tests and expressed as ‘greater than’^58^. All values less than LOQ are treated as zero.

We quantify individual compound risk (Table 2 and Supplementary Table 2) as the average of concentrations for a given compound divided by its respective LD_50_ and multiplied by its site detection frequency^62^. To calculate the dominance of individual compounds to the mixture risk, we determine the MCR of each pollen sample as the additive toxicity-weighted concentration of the mixture (TWC_mix_) divided by the highest toxicity-weighted concentration of a single mixture component (max(TWC_*i*_))^63^

$${\rm{MCR}}=\frac{{{\rm{TWC}}}_{{\rm{mix}}}}{\max \left({{\rm{TWC}}}_{i}\right)}$$

When MCR = 1, risk comes from a single compound; thus, the MCR represents the factor by which the pesticide mixture is riskier than the single most risky compound.

### Statistical analyses

**Extended Data Fig. 9:**
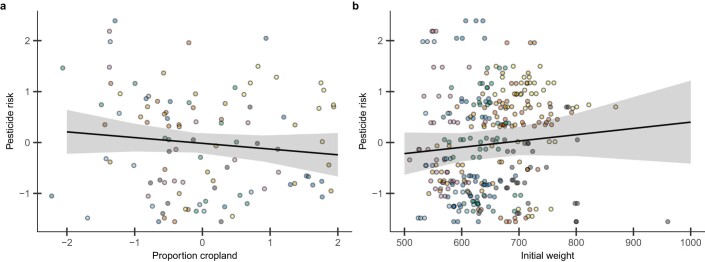
Risk is not correlated with (a) proportion cropland or (b) initial colony weight. Fitted lines and 95% confidence intervals (**a,b**) are estimated based on linear mixed effects models. Point colours correspond to country colours in Fig. 1a, S5a and Extended Data Fig. 5a.

We tested whether pesticide risk (TWC_mix_) interacts with crop type and proportion cropland to affect our measures of colony performance (total colony production, weight gain, maximum weight and queen production). Given a strong right skew, we log-transformed (ln(*x* + 0.1)) risk values. We centred risk and cropland values to aid the interpretation of interaction terms. For weight gain, we specified an LMM with risk, crop type, proportion cropland and their interactions as fixed effects and with site nested in country as a random effect. We specified a GLMM with a negative binomial error distribution for overdispersed count data of total colony production (dispersion ratio = 54.98; *P* < 0.001). We used the same fixed and random effect structure as above. We analysed two more measures of colony performance: maximum weight and queen cocoon production (total of intact and eclosed queen cocoons). We specified an LMM as above with weight log-transformed because it improved diagnostics of model residuals and our results are qualitatively similar if weight is untransformed (Extended Data Table 1). We specified a GLMM as above for queen cocoon production and with a single, constant zero-inflation parameter (Extended Data Table 1). We included initial colony weight (*g*_initial_) as a covariate in the above models to account for variation in colony starting conditions. Models showed little multicollinearity (VIF range 1.03–3.28 across all models) and we confirmed that risk and proportion of cropland were independent by means of an LMM with the country as a random effect (marginal *R*^2^ (*R*^2^m) = 0.02; *χ*^2^ = 1.38, *P* = 0.24; Extended Data Fig. 9a). We also confirmed that risk was independent of initial colony weight by means of an LMM with the country as a random effect (*R*^2^m = 0.01; *χ*^2^ = 1.22, *P* = 0.27; Extended Data Fig. 9b). We performed analyses and data visualization using R v.4.1.1. We constructed LMMs with the lme4 package^64^ and GLMMs with the glmmTMB package^65^. We report *R*^2^ values calculated following the methods of ref. ^66^. We estimated marginal means with the emmeans package^67^. We estimated interaction slopes with the interactions package^68^ and evaluated models for overdispersion, normality and multicollinearity using diagnostic functions in the performance package^69^ and the DHARMa package^70^.

### Reporting summary

Further information on research design is available in the Nature Portfolio Reporting Summary linked to this article.

## Online content

Any methods, additional references, Nature Portfolio reporting summaries, source data, extended data, supplementary information, acknowledgements, peer review information; details of author contributions and competing interests; and statements of data and code availability are available at 10.1038/s41586-023-06773-3.

### Supplementary information

Supplementary Information Supplementary Table 1, a complete list of pesticides screened in the bumble bee colony pollen stores; Table 2, a complete list of pesticides quantified in the colony pollen stores; and Table 3, a complete list of colonies not included in the analysis.

Reporting Summary

Peer Review File

## Figures and Tables

**Extended Data Table 1:**
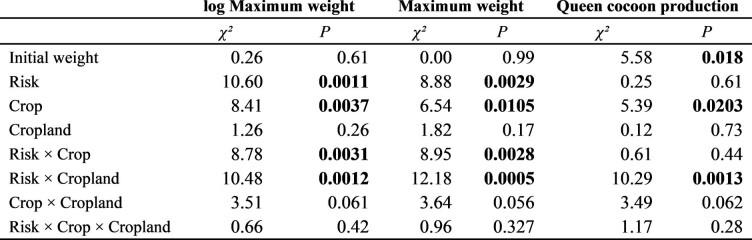
Additional colony performance results

Colony log-transformed maximum weight, maximum weight and queen cocoon production in relation to initial colony weight, pesticide risk (sum of toxicity-weighted pesticide concentrations: see Methods), crop type (oilseed rape, apple), the proportion of cropland in the surrounding landscape (1 km radius) and their interactions.

**Extended Data Table 2:**
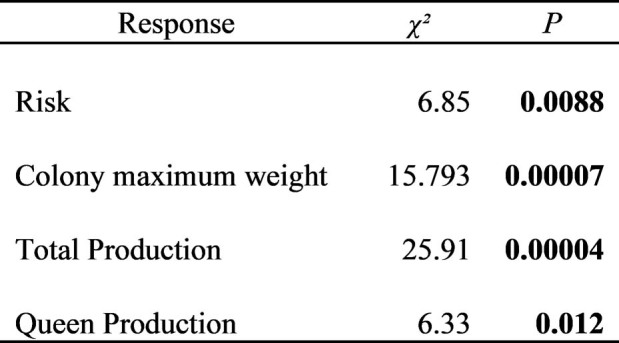
Comparisons of colony performance between risk groups

Colony risk between colonies’ SPG status and maximum weight, total production and queen production between low-risk (25^th^ precentile of risk) and high-risk (90^th^ percentile of risk) colony groups. Results are from linear (risk and weight) and generalized linear mixed effects models. See Extended Data Fig. 6 for details on SPG and risk groups.

## Data Availability

The datasets analysed for the current study are available through the PoshBee project (Deliverable D1.6 Database of field records), the Pesticide Properties Database and through figshare: 10.6084/m9.figshare.24235573.
