# Supplementary material for: Pesticide use negatively affects bumble bees across European landscapes
Source: Nature. 2023 Nov 29;628(8007):355–8. doi: 10.1038/s41586-023-06773-3 (PMC11006599; doi:10.1038/s41586-023-06773-3)
Supplement: Supplementary file 1 — Supplementary Table 1, a complete list of pesticides screened in the bumble bee colony pollen stores; Table 2, a complete list of pesticides quantified in the colony pollen stores; and Table 3, a complete list of colonies not included in the analysis. [file 41586_2023_6773_MOESM1_ESM.pdf]

nature portfolio

https://doi.org/10.1038/s41586-023-06773-3

Supplementary information

Pesticide use negatively affects bumble bees across European landscapes

In the format provided by the authors and unedited

SUPPLEMENTARY INFORMATION

Pesticide use negatively affects bumble bees across European landscapes

Charlie C. Nicholson (charlie.nicholson@biol.lu.se), Jessica Knapp (jessica.knapp@biol.lu.se), Tomasz Kiljanek, Matthias Albrecht, Marie-Pierre Chauzat, Cecilia Costa, Pilar De la Rúa, Alexandra-Maria Klein, Marika Mänd, Simon G. Potts, Oliver Schweiger, Irene Bottero, Elena Cini, Joachim R. de Miranda, Gennaro Di Prisco, Christophe Dominik, Simon Hodge, Vera Kaunath, Anina Knauer, Marion Laurent, Vicente Martínez-López, Piotr Medrzycki, Maria Helena Pereira-Peixoto, Risto Raimets, Janine M. Schwarz, Deepa Senapathi, Giovanni Tamburini, Mark J.F. Brown, Jane C. Stout, Maj Rundlöf (maj.rundlof@biol.lu.se)

Contents

SUPPLEMENTARY TABLES 2

Supplementary Table 1 Complete list of pesticides screened in the colony pollen stores 2

Supplementary Table 2 Complete list of pesticides quantified in the colony pollen stores. 11

Supplementary Table 3 Colonies not included in the analysis 15

SUPPLEMENTARY TABLES

Supplementary Table 1 Complete list of pesticides screened in the colony pollen stores. Pesticide identity, category of use (a: acaricide, v: veterinary drugs, f: fungicide, h: herbicide, i: insecticide, r: plant growth regulator, c: contaminant), pesticide approval status under Reg. (EC) No 1107/2009.

Compound	Category	Approval under Reg. (EC) No 1107/2009 (accessed August 11, 2020)	Approval under Reg. (EC) No 1107/2009 (accessed July 12, 2023)
1-Naphthylacetamide (1-NAD)	R	Approved	Approved
2,4-D	H	Approved	Approved
6-chloro-4-hydroxy-3-phenyl-pyridazine	H	Approved	Approved
6-hydroxy bentazone	H	Approved	Approved
Acequinocyl	A	Approved	Approved
Acetamiprid	I	Approved	Approved
Acetochlor	H	Not approved	Not approved
Acrinathrin	V	Approved	Not approved
Aldrin	I	Not approved	Not approved
alpha-Endosulfan	I	Not approved	Not approved
alpha-HCH	I	Not approved	Not approved
Amidosulfuron	H	Approved	Approved
Asulam	H	Pending	Pending
Azinphos-ethyl	I	Not approved	Not approved
Azinphos-methyl	I	Not approved	Not approved
Azoxystrobin	F	Approved	Approved
Bentazone	H	Approved	Approved
beta-Endosulfan	I	Not approved	Not approved
beta-HCH	I	Not approved	Not approved
Bifenazate	A	Approved	Approved
Bifenox	H	Approved	Approved
Bifenthrin	I	Not approved	Not approved
Bixafen	F	Approved	Approved
Boscalid	F	Approved	Approved

Bromopropylate	A	Not approved	Not approved
Bromoxynil	H	Approved	Not approved
Bupirimate	F	Approved	Approved
Carbaryl	I	Not approved	Not approved
Carbendazim	F	Not approved	Not approved
Carbetamide	H	Approved	Not approved
Carboxin	F	Approved	Not approved
Carfentrazone-ethyl	H	Approved	Approved
Chlorantraniliprole	I	Approved	Approved
Chlorfenvinphos	I	Not approved	Not approved
Chloridazon	H	Not approved	Not approved
Chlorothalonil	F	Not approved	Not approved
Chlorotoluron	H	Approved	Approved
Chlorpropham	R	Not approved	Not approved
Chlorpyrifos	I	Not approved	Not approved
Chlorpyrifos-methyl	I	Not approved	Not approved
Chlorsulfuron	H	Not approved	Not approved
cis-Chlordane	I	Not approved	Not approved
cis-Heptachlor epoxide	I	Not approved	Not approved
Clethodim	H	Approved	Approved
Clofentezine	A	Approved	Approved
Clomazone	H	Approved	Approved
Clothianidin	I	Not approved	Not approved
Coumaphos	V	Not approved	Not approved
Cyantraniliprole	I	Approved	Approved
Cyazofamid	F	Approved	Approved
Cycloxydim	H	Approved	Approved
Cyflufenamid	F	Approved	Approved
Cyfluthrin (sum of isomers)	I	Not approved	Not approved
Cymiazol	V		-
Cymoxanil	F	Approved	Approved
Cypermethrin (sum of isomers)	I	Approved	Approved
Cyproconazole	F	Approved	Not approved
Cyprodinil	F	Approved	Approved

Deltamethrin	I	Approved	Approved
Desmedipham	H	Not approved	Not approved
Diazinon	I	Not approved	Not approved
Dichlorprop-P	H	Approved	Approved
Dieldrin	I	Not approved	Not approved
Difenoconazole	F	Approved	Approved
Diflubenzuron	I	Approved	Not approved
Diflufenican	H	Approved	Approved
Dimethachlor	H	Approved	Approved
Dimethoate	I	Not approved	Not approved
Dimethomorph	F	Approved	Approved
Dimoxystrobin	F	Approved	Approved
Dithianon	F	Approved	Approved
Dodine	F	Approved	Approved
Endosulfan sulfate	I	Not approved	Not approved
Endrin	I	Not approved	Not approved
Epoxiconazole	F	Not approved	Not approved
Esfenvalerate (Fenvalerate)	I	Approved	Approved
Ethametsulfuron-methyl		Pending	Not approved
Ethofumesate	H	Approved	Approved
Ethoprophos	I	Not approved	Not approved
Etofenprox	I	Approved	Approved
Etoxazole	A	Approved	Approved
Famoxadone	F	Approved	Not approved
Fenazaquin	A	Approved	Approved
Fenbuconazole	F	Approved	Not approved
Fenhexamid	F	Approved	Approved
Fenitrothion	I	Not approved	Not approved
Fenoxaprop-P-ethyl	H	Approved	Approved
Fenoxycarb	I	Approved	Not approved
Fenpropidin	F	Approved	Approved
Fenpropimorph	F	Not approved	Not approved
Fenpyroximate	A	Approved	Approved
Fenthion	I	Not approved	Not approved

Fenthion-sulfone	I	Not approved	Not approved
Fenthion-sulfoxide	I	Not approved	Not approved
Fipronil	I	Not approved	Not approved
Fipronil-carboxamide	I	Not approved	Not approved
Fipronil-desulfinyl	I	Not approved	Not approved
Fipronil-sulfide	I	Not approved	Not approved
Fipronil-sulfone	I	Not approved	Not approved
Flazasulfuron	H	Approved	Approved
Flonicamid	I	Approved	Approved
Florasulam	H	Approved	Approved
Fluazifop-P-butyl	H	Approved	Approved
Fluazinam	F	Approved	Approved
Fludioxonil	F	Approved	Approved
Flufenacet	H	Approved	Approved
Fluopyram	F	Approved	Approved
Flupyradifurone	I	Approved	Approved
Fluquinconazole	F	Approved	Not approved
Flurochloridone	H	Approved	Approved
Fluroxypyr	H	Approved	Approved
Fluroxypyr-meptyl	H	Approved	Approved
Flurprimidol	R	Not approved	Not approved
Flusilazole	F	Not approved	Not approved
Flutriafol	F	Approved	Not approved
Fluxapyroxad	F	Approved	Approved
Foramsulfuron	H	Approved	Approved
Gibberellin A4	R	Approved	Approved
HCB	I	Not approved	Not approved
Heptachlor	I	Not approved	Not approved
Heptenophos	I	Not approved	Not approved
Hexythiazox	A	Approved	Approved
Hymexazol	F	Approved	Approved
Imazalil	F	Approved	Approved
Imidacloprid	I	Approved	Not approved
Imidacloprid-olefin	I	Approved	Not approved

Imidacloprid-urea	I	Approved	Not approved
Indolylbutyric acid (IBA)	R	Approved	Approved
Indoxacarb	I	Approved	Not approved
Iodosulfuron-methyl-sodium	H	Approved	Approved
Ipconazole	F	Approved	Not approved
Iprodione	F	Not approved	Not approved
Isoproturon	H	Not approved	Not approved
Isopyrazam	F	Approved	Not approved
Isoxaflutole	H	Approved	Approved
Kresoxim-methyl	F	Approved	Approved
lambda-Cyhalothrin	I	Approved	Approved
Lenacil	H	Approved	Approved
Lindane	I	Not approved	Not approved
Linuron	H	Not approved	Not approved
Malathion	I	Approved	Approved
Mandipropamid	F	Approved	Approved
MCPA	H	Approved	Approved
MCPB	H	Approved	Approved
Mecoprop-P	H	Approved	Approved
Mepanipyrim	F	Approved	Approved
Mesosulfuron-methyl	H	Approved	Approved
Mesotrione	H	Approved	Approved
Metaflumizone	I	Approved	Approved
Metalaxyl-M (Metalaxyl)	F	Approved	Approved
Metamitron	H	Approved	Approved
Metazachlor	H	Approved	Approved
Metconazole	F	Approved	Approved
Methidathion	I	Not approved	Not approved
Methiocarb	I	Not approved	Not approved
Methiocarb sulfoxide	I	Not approved	Not approved
Methiocarb sulfone	I	Not approved	Not approved
Methoxychlor	I	Not approved	Not approved
Methoxyfenozide	I	Approved	Approved
Metrafenone	F	Approved	Approved

Metribuzin	H	Approved	Approved
Metsulfuron-methyl	H	Approved	Approved
Mevinphos	I	Not approved	Not approved
Myclobutanil	F	Approved	Not approved
N-2,4-Dimethylphenyl-formamide (DMF)	V		-
N-2,4-Dimethylphenyl-N’-methylformamide (DMPF)	V		-
Napropamide	H	Approved	Approved
Nicosulfuron	H	Approved	Approved
Nitenpyram	I	Not approved	Not approved
Novaluron	I	Not approved	Not approved
Omethoate	I	Not approved	Not approved
Oxychlordane	I	Not approved	Not approved
Oxyfluorfen	H	Approved	Approved
o,p’-DDT	I	Not approved	Not approved
p,p’-DDD	I	Not approved	Not approved
p,p’-DDE	I	Not approved	Not approved
p,p’-DDT	I	Not approved	Not approved
Paclobutrazol	F	Approved	Approved
Parathion-ethyl	I	Not approved	Not approved
Parathion-methyl	I	Not approved	Not approved
PCB 101	C	-	-
PCB 138	C	-	-
PCB 153	C	-	-
PCB 180	C	-	-
PCB 28	C	-	-
PCB 52	C	-	-
Penconazole	F	Approved	Approved
Pencycuron	F	Approved	Not approved
Pendimethalin	H	Approved	Approved
Penthiopyrad	F	Approved	Approved
Permethrin (sum of isomers)	I	Not approved	Not approved
Pethoxamid	H	Approved	Approved
Phenmedipham	H	Approved	Approved
Phosalone	I	Not approved	Not approved

Phosmet	I	Approved	Not approved
Phoxim	I	Not approved	Not approved
Picoxystrobin	F	Not approved	Not approved
Pirimicarb	I	Approved	Approved
Pirimicarb-desmethyl	I	Approved	Approved
Pirimiphos-ethyl	I	Not approved	Not approved
Pirimiphos-methyl	I	Approved	Approved
Prochloraz	F	Approved	Not approved
Profenofos	I	Not approved	Not approved
Propamocarb	F	Approved	Approved
Propaquizafop	H	Approved	Approved
Propargite	A	Not approved	Not approved
Propiconazole	F	Not approved	Not approved
Propoxur	I	Not approved	Not approved
Propoxycarbazone-sodium	H	Approved	Approved
Propyzamide	H	Approved	Approved
Proquinazid	F	Approved	Approved
Prosulfocarb	H	Approved	Approved
Prothioconazole	F	Approved	Approved
Prothioconazole-desthio	F	Approved	Approved
Pymetrozine	I	Not approved	Not approved
Pyraclostrobin	F	Approved	Approved
Pyrazophos	F	Not approved	Not approved
Pyridate	H	Approved	Approved
Pyrimethanil	F	Approved	Approved
Pyriproxyfen	I	Approved	Approved
Quinmerac	H	Approved	Approved
Quinoclamine	H	Not approved	Not approved
Quinoxyfen	F	Not approved	Not approved
Quizalofop-P-ethyl	H	Approved	Approved
Quizalofop-P-tefuryl	H	Approved	Approved
Resmethrin	I	Not approved	Not approved
Rimsulfuron	H	Approved	Approved
Silthiofam	F	Approved	Approved

S-Metolachlor	H	Approved	Approved
Spinosad (mix of Spinosyn A & D)	I	Approved	Approved
Spirodiclofen	I	Not approved	Not approved
Spirotetramat	I	Approved	Approved
Spirotetramat-enol	I	Approved	Approved
Spirotetramat-enol glucoside	I	Approved	Approved
Spirotetramat-keto hydroxy	I	Approved	Approved
Spiroxamine	F	Approved	Approved
Sulcotrione	H	Approved	Approved
Sulfosulfuron	H	Approved	Approved
Sulfoxaflor	I	Approved	Approved
tau-Fluvalinate	I	Approved	Approved
Tebuconazole	F	Approved	Approved
Tebufenozide	I	Approved	Approved
Tebufenpyrad	A	Approved	Approved
Teflubenzuron	I	Not approved	Not approved
Tefluthrin	I	Approved	Approved
Tembotrione	H	Approved	Approved
Tepraloxydim	H	Not approved	Not approved
Terbuthylazine	H	Approved	Approved
Tetraconazole	F	Approved	Approved
Tetramethrin	I	Not approved	Not approved
Thiacloprid	I	Not approved	Not approved
Thiacloprid-amide	I	Not approved	Not approved
Thiamethoxam	I	Not approved	Not approved
Thifensulfuron-methyl	H	Approved	Approved
Thiophanate-methyl	F	Approved	Not approved
Tralkoxydim	H	Not approved	Not approved
trans-Chlordane	I	Not approved	Not approved
trans-Heptachlor epoxide	I	Not approved	Not approved
Triadimefon	F	Not approved	Not approved
Triadimenol	F	Not approved	Not approved
Triazophos	I	Not approved	Not approved
Tribenuron-methyl	H	Approved	Approved

Trifloxystrobin	F	Approved	Approved
Triflusulfuron-methyl	H	Approved	Approved
Trinexapac-ethyl	R	Approved	Approved
Triticonazole	F	Approved	Approved
Vinclozolin	F	Not approved	Not approved

Supplementary Table 2 Complete list of pesticides quantified in the colony pollen stores. Pesticide identity, category (I: insecticide, F: fungicide, H: herbicide, A: acaricide, R: plant growth regulator), chemical group, crops in which the pesticide was quantified (apple: APP; oilseed rape: OSR), frequency of quantification (number of sites out of 106 sites), toxicity (average acute oral and contact LD50 for *Apis mellifera* adults, considering worst case from 24, 48 and 72 hour values, µg/bee [46]), limit of quantification (LOQ; µg/kg), concentrations (mean, median, 90th percentile; µg/kg), and compound pesticide risk (see Methods).

Compound	Chemical group	Focal crop detected	Quantification frequency	LD$_{50}$ oral	LD$_{50}$ contact	LD$_{50}$ mean	LOQ	Concentration mean	Concentration median	Concentration 90th percentile	Pesticide risk
Indoxacarb (I)	Oxadiazine	APP / OSR	17 (16%)	0.232	0.08	0.156	5	1310	57	3380	1430
Spinosad (I)	Spinosyn	APP	2 (2%)	0.057	0.004	0.03	5	658	658	1170	434
Chlorpyrifos-Ethyl (I)	Organophosphate	APP / OSR	9 (8%)	0.15	0.068	0.109	5	282	13.9	561	233
Deltamethrin (I)	Pyrethroid	APP	2 (2%)	0.07	0.002	0.036	5	68.8	68.8	117	38.5
Dimethoate (I)	Organophosphate	APP / OSR	11 (10%)	0.1	0.1	0.1	1	31.0	15.4	77.3	34.1
Imidacloprid (I)	Neonicotinoid	APP / OSR	9 (8%)	0.004	0.081	0.042	1	9.49	8.1	17.5	20.2
Cyfluthrin (I)	Pyrethroid	APP	1 (1%)	0.05	0.001	0.026	1	41.5	41.5	41.5	16.3
Dithianon (F)	Quinone	APP / OSR	25 (24%)	25.4	100	62.7	50	3300	244	12900	12.6
Etofenprox (I)	Pyrethroid	APP	3 (3%)	0.366	0.038	0.202	5	61.9	47.5	91.9	9.19
Chlorpyrifos-Methyl (I)	Organophosphate	APP / OSR	4 (4%)	0.177	0.148	0.162	5	36.9	16.6	80.9	9.08
Phosmet (I)	Organophosphate	APP	2 (2%)	0.37	0.22	0.295	50	123	123	175	8.35
Methiocarb (I)	Carbamate	APP / OSR	4 (4%)	0.08	0.23	0.155	5	16.1	15.6	23	4.16
Cypermethrin (I)	Pyrethroid	APP / OSR	4 (4%)	0.172	0.023	0.097	1	7.70	5	15	3.16
Acetamiprid (I)	Neonicotinoid	APP / OSR	29 (27%)	14.53	8.09	11.31	1	127	18.4	476	3.15
Trifloxystrobin (F)	Strobilurin	APP / OSR	27 (25%)	110	100	105	1	1100	32.8	3450	2.73
Pyrimethanil (F)	Anilinopyrimidine	APP / OSR	27 (25%)	100	100	100	5	887	121	2640	2.31
Thiacloprid (I)	Neonicotinoid	APP / OSR	40 (38%)	17.32	38.82	28.07	1	106	17.8	260	1.47
Boscalid (F)	Carboxamide	APP / OSR	48 (45%)	166	200	183	1	404	10.7	1090	1.04
Penconazole (F)	Triazole	APP / OSR	8 (8%)	11.2	3	7.1	10	85.3	23.8	237	0.961
Fluazinam (F)	Pyridine; Aminopyridine; Chloropyridine	APP / OSR	16 (15%)	100	200	150	1	866	15.3	3270	0.924
Fenhexamid (F)	Hydroxyanilide	APP / OSR	9 (8%)	102.07	200	151.035	10	1100	119	2590	0.653
Pirimicarb (I)	Carbamate	APP / OSR	8 (8%)	4	17.8	10.9	5	71.0	17.4	150	0.586
Myclobutanil (F)	Triazole	APP	8 (8%)	33.9	33.9	33.9	5	245	107	618	0.579

1-Naphthylacetamide (R)	Auxin	APP	7 (7%)	NA	100	100	5	678	215	1900	0.543
Kresoxim-Methyl (F)	Strobilurin	APP	9 (8%)	110	100	105	1	806	29	2620	0.537
Flonicamid (I)	Pyridine	APP	18 (17%)	100	100	100	10	346	128	602	0.484
Cyprodinil (F)	Anilinopyrimidine	APP / OSR	14 (13%)	112.5	784	448.25	5	1210	38	1190	0.460
Pyraclostrobin (F)	Strobilurin	APP / OSR	23 (22%)	110	100	105	1	209	4.3	689	0.438
Tau-Fluvalinate (I)	Pyrethroid	APP / OSR	8 (8%)	12.6	12	12.3	1	59.4	6.4	139	0.386
Fluxapyroxad (F)	Pyrazolium	APP / OSR	35 (33%)	110.9	100	105.45	1	116	8.8	376	0.373
Heptachlor Epoxide Isomer B (I)	Organochloride	OSR	2 (2%)	NA	0.526	0.526	5	8.45	8.45	10.7	0.321
Difenoconazole (F)	Triazole	APP / OSR	31 (29%)	177	100	138.5	1	127	12.8	430	0.274
Thiophanate-Methyl (F)	Benzimidazole	APP / OSR	26 (25%)	114.7	100	107.35	1	108	18	410	0.252
Penthiopyrad (F)	Carboxamide	APP / OSR	14 (13%)	500	500	500	1	574	17.3	1990	0.161
Fludioxonil (F)	Phenylpyrrole	APP / OSR	12 (11%)	100	100	100	5	124	73.8	317	0.149
Imazalil (F)	Imidazole	OSR	6 (6%)	35.1	39	37.05	10	82.5	56.6	178	0.134
Tebufenozide (I)	Diacylhydrazine	APP	6 (6%)	100	234	167	10	332	95.8	848	0.119
Iprodione (F)	Dicarboximide	APP	2 (2%)	100	100	100	50	522	522	892	0.104
Terbuthylazine (H)	Triazine	APP / OSR	21 (20%)	22.6	32	27.3	5	25.8	10.9	73.4	0.104
Carbendazim (F)	Benzimidazole; Carbamate	APP / OSR	11 (10%)	100	50	75	1	35.7	8.4	88.7	0.0951
Azinphos-Methyl (I)	Organophosphorus	APP	1 (1%)	NA	0.42	0.42	1	3.90	3.9	3.9	0.0929
Chlorothalonil (F)	Chloronitrile	APP / OSR	1 (1%)	40	40	40	10	34.4	13.6	70.6	0.0859
Esfenvalerate (I)	Pyrethroid	APP	2 (2%)	0.21	0.06	0.135	1	1.10	1.1	1.1	0.0815
Famoxadone (F)	Oxazole	APP / OSR	10 (9%)	1	25	13	1	52.2	52.2	79.9	0.0803
Dimoxystrobin (F)	Strobilurin	APP / OSR	10 (9%)	79.4	100	89.7	1	66.7	60.8	129	0.0743
Pendimethalin (H)	Dinitroaniline	APP / OSR	66 (62%)	101.2	100	100.6	1	10.8	5.4	18.6	0.0690
Fluopyram (F)	Benzamide, pyramide	APP / OSR	33 (31%)	102.3	100	101.15	1	21.0	4.3	30.6	0.0663
Prothioconazole-Desthio (F)	Triazolinthione	APP / OSR	24 (23%)	71	100	85.5	10	23.2	16.2	42.4	0.0624
Tebuconazole (F)	Triazole	APP / OSR	30 (28%)	83.05	200	141.525	5	29.8	14.4	89.6	0.0611
Azoxystrobin (F)	Strobilurin	APP / OSR	30 (28%)	25	200	112.5	1	19.2	2.6	69.2	0.0494
Proquinazid (F)	Quinazolinone	APP	3 (3%)	125	197	161	5	237	228	341	0.0441
Gibberellin A4 (R)	Diterpenoid acids	APP / OSR	2 (2%)	84	100	92	50	169	169	190	0.0367
Pirimicarb-Desmethyl (I)	Carbamate	APP / OSR	6 (6%)	4	17.8	10.9	1	6.08	3.15	12.7	0.0335
Trans-Chlordane (I)	Organochloride	OSR	1 (1%)	NA	0.6	0.6	1	2.00	2	2	0.0333
2,4-D (H)	Alkylchlorophenoxy	APP	2 (2%)	94	100	97	50	160	160	227	0.0330

Prosulfocarb (H)	Thiocarbamate	APP / OSR	18 (17%)	103.4	80	91.7	5	16.1	7.75	40	0.0298
Acequinocyl (A)	Unclassified	APP	2 (2%)	100	100	100	100	148	148	185	0.0295
Spirotetramat-Enol (I)	Tetramic acid	APP	1 (1%)	107.3	100	103.65	5	238	238	238	0.0230
Dimethomorph (F)	Morpholine	APP / OSR	16 (15%)	32.4	102	67.2	1	9.49	7.35	17.9	0.0226
Diflufenican (H)	Carboxamide	OSR	2 (2%)	107.4	100	103.7	1	108	108	190	0.0208
Cyflufenamid (F)	Amidoxine	APP	6 (6%)	100	100	100	5	31.8	10	80.2	0.0191
Thiacloprid-Amide (I)	Neonicotinoid	APP / OSR	4 (4%)	17.32	38.82	28.07	5	13.3	8.65	23	0.0190
Tetraconazole (F)	Triazole	APP / OSR	5 (5%)	130	63	96.5	5	33.5	10.9	77	0.0174
Fenbuconazole (F)	Triazole	APP	1 (1%)	5.2	5.5	5.35	5	8.70	8.7	8.7	0.0163
Methoxyfenozide (I)	Diacylhydrazine	APP	4 (4%)	2000	100	1050	50	359	214	740	0.0137
Fluroxypyr-Meptyl (H)	Pyridine	APP	1 (1%)	100	100	100	50	131	131	131	0.0131
S-Metolachlor (H)	Chloroacetamide	APP / OSR	13 (12%)	85	200	142.5	1	11.3	2.3	8.56	0.0103
Fenpropimorph (F)	Morpholine	APP / OSR	14 (13%)	95.6	100	97.8	1	6.21	2.05	8.46	0.00890
Bupirimate (F)	Pyrimidine	APP / OSR	10 (9%)	200	50	125	1	10.6	8.4	22.5	0.00851
Quinoclamine (H)	unclassified	OSR	1 (1%)	100	75	87.5	5	46.4	46.4	46.4	0.00530
Fenpyroximate (A)	Pyrazolium	APP	2 (2%)	118.5	15.8	67.15	1	16.0	16	25.6	0.00475
Chlorantraniliprole (I)	Anthranilic diamide	APP / OSR	3 (3%)	104.1	100	102.05	1	16.0	14.2	27.4	0.00470
Pyriproxyfen (I)	Unclassified	APP	3 (3%)	100	74	87	5	12.9	9.7	20.5	0.00444
Spirotetramat (I)	Tetramic acid	APP	3 (3%)	107.3	100	103.65	1	12.1	4.7	23.7	0.00351
Mcpa (H)	Aryloxyalkanoic acid	APP	1 (1%)	200	200	200	50	51.7	51.7	51.7	0.00258
Metamitron (H)	Triazinone	OSR	2 (2%)	97.2	100	98.6	10	12.0	12	12.2	0.00243
Mepanipyrim (F)	Anilinopyrimidine	APP / OSR	2 (2%)	51	100	75.5	5	8.45	8.45	9.85	0.00224
Lenacil (H)	Uracil	OSR	2 (2%)	206.2	227.2	216.7	1	20.8	20.8	35.7	0.00192
Fenpropidin (F)	unclassified	APP / OSR	2 (2%)	10	46	28	1	2.55	2.55	3.47	0.00182
Prochloraz (F)	Imidazole	OSR	1 (1%)	101	141.3	121.15	5	20.4	20.4	20.4	0.00168
Epoxiconazole (F)	Triazole	OSR	1 (1%)	83	100	91.5	10	13.3	13.3	13.3	0.00145
Clethodim (H)	Cyclohexanedione	APP	1 (1%)	43	51	47	5	6.80	6.8	6.8	0.00145
Hexythiazox (A)	Carboxamide	APP	3 (3%)	112	200	156	1	7.13	7	10.4	0.00137
Propyzamide (H)	Benzamide	APP / OSR	2 (2%)	100	136	118	5	7.10	7.1	7.34	0.00120
Cyproconazole (F)	Conazole	OSR	1 (1%)	100	100	100	5	12.0	12	12	0.00120
Clomazone (H)	Isoxazolidinone	APP / OSR	4 (4%)	76.33	89.5	82.915	1	1.88	1.65	2.81	0.000905
Spirodiclofen (A)	Tetronic acid	APP / OSR	2 (2%)	196	200	198	1	6.10	6.1	6.98	0.000616
Bentazone (H)	Benzothiazinone	OSR	2 (2%)	200	200	200	1	4.85	4.85	5.29	0.000485

Bixafen (F)	Pyrazolium	OSR	1 (1%)	100	121.4	110.7	5	5.00	5	5	0.000452
Metalaxyl (F)	Phenylamide	APP	1 (1%)	269	200	234.5	5	9.60	9.6	9.6	0.000409
Pencycuron (F)	Phenylurea	APP	2 (2%)	98.5	100	99.25	1	1.70	1.7	1.78	0.000343
Mandipropamid (F)	Mandelamide	OSR	1 (1%)	200	200	200	5	5.10	5.1	5.1	0.000255
Chlorotoluron (H)	Urea	APP	3 (3%)	100.1	200.2	150.15	1	1.17	1.2	1.2	0.000233
6-Hydroxy Bentazon (H)	Benzothiazinone	OSR	1 (1%)	200	200	200	1	3.60	3.6	3.6	0.000180
Flufenacet (H)	Anilide	OSR	1 (1%)	100	109.2	104.6	1	1.50	1.5	1.5	0.000143
Amidosulfuron (H)	Sulfonylurea	OSR	1 (1%)	109.2	100	104.6	1	1.10	1.1	1.1	0.000105
Isopyrazam (F)	Pyrazole	OSR	1 (1%)	192.3	200	196.15	1	1.40	1.4	1.4	0.0000714
Chlorpropham (H)	Carbamate	OSR	1 (1%)	505	96.1	300.55	1	1.30	1.3	1.3	0.0000433

Supplementary Table 3 Colonies not included in the analysis. From an initial 384 colonies deployed, 316 provide information for analysis, while 68 colonies could not be included in the analyses due to either colony loss (5 colonies) or insufficient pollen store material for pesticide quantification (63 colonies).

Country	SiteID	ColonyID	Reason	Data missing
GBR	GBR_APP_14	All colonies	Colonies damaged	weight and production measures
GER	GER_OSR_07	GER_OSR_07_B2	Colony lost to a raccoon attack	weight and production measures
ESP	ESP_OSR_01	ESP_OSR_01_B2	Colony lost to tractor runover	weight and production measures
CHE	CHE_APP_12	All colonies	Insufficient colony pollen for sample	pesticide residues, palynology
CHE	CHE_APP_15	All colonies	Insufficient colony pollen for sample	pesticide residues, palynology
ESP	ESP_APP_11	All colonies	Insufficient colony pollen for sample	pesticide residues, palynology
ESP	ESP_APP_12	All colonies	Insufficient colony pollen for sample	pesticide residues, palynology
ESP	ESP_OSR_06	All colonies	Insufficient colony pollen for sample	pesticide residues, palynology
EST	EST_APP_11	All colonies	Insufficient colony pollen for sample	pesticide residues, palynology
EST	EST_APP_14	All colonies	Insufficient colony pollen for sample	pesticide residues, palynology
EST	EST_OSR_01	All colonies	Insufficient colony pollen for sample	pesticide residues, palynology
EST	EST_OSR_04	All colonies	Insufficient colony pollen for sample	pesticide residues, palynology
EST	EST_OSR_06	All colonies	Insufficient colony pollen for sample	pesticide residues, palynology
GBR	GBR_APP_10	All colonies	Insufficient colony pollen for sample	pesticide residues, palynology
GBR	GBR_APP_16	All colonies	Insufficient colony pollen for sample	pesticide residues, palynology
IRE	IRE_APP_11	All colonies	Insufficient colony pollen for sample	pesticide residues, palynology
IRE	IRE_APP_13	All colonies	Insufficient colony pollen for sample	pesticide residues, palynology
IRE	IRE_APP_15	All colonies	Insufficient colony pollen for sample	pesticide residues, palynology
IRE	IRE_APP_16	All colonies	Insufficient colony pollen for sample	pesticide residues, palynology
IRE	IRE_OSR_01	All colonies	Insufficient colony pollen for sample	pesticide residues, palynology
IRE	IRE_OSR_06	All colonies	Insufficient colony pollen for sample	pesticide residues, palynology
IRE	IRE_OSR_07	All colonies	Insufficient colony pollen for sample	pesticide residues, palynology
ITA	ITA_OSR_02	All colonies	Insufficient colony pollen for sample	pesticide residues, palynology
SWE	SWE_APP_12	All colonies	Insufficient colony pollen for sample	pesticide residues, palynology
