# Supplementary material for: Pesticide use negatively affects bumble bees across European landscapes
Source: Nature. 2023 Nov 29;628(8007):355–8. doi: 10.1038/s41586-023-06773-3 (PMC11006599; doi:10.1038/s41586-023-06773-3)
Supplement: Supplementary file 2 — Reporting Summary [file 41586_2023_6773_MOESM2_ESM.pdf]

nature portfolio

Corresponding author(s): Charlie C. Nicholson, Jessica Knapp, Maj Rundlöf

Last updated by author(s): Sep 25, 2023

Reporting Summary

Nature Portfolio wishes to improve the reproducibility of the work that we publish. This form provides structure for consistency and transparency in reporting. For further information on Nature Portfolio policies, see our Editorial Policies and the Editorial Policy Checklist.

Statistics

For all statistical analyses, confirm that the following items are present in the figure legend, table legend, main text, or Methods section.

n/a	Confirmed	
☐	☒	The exact sample size (*n*) for each experimental group/condition, given as a discrete number and unit of measurement
☐	☒	A statement on whether measurements were taken from distinct samples or whether the same sample was measured repeatedly
☐	☒	The statistical test(s) used AND whether they are one- or two-sided<br>*Only common tests should be described solely by name; describe more complex techniques in the Methods section.*
☐	☒	A description of all covariates tested
☐	☒	A description of any assumptions or corrections, such as tests of normality and adjustment for multiple comparisons
☐	☒	A full description of the statistical parameters including central tendency (e.g. means) or other basic estimates (e.g. regression coefficient) AND variation (e.g. standard deviation) or associated estimates of uncertainty (e.g. confidence intervals)
☐	☒	For null hypothesis testing, the test statistic (e.g. *F*, *t*, *r*) with confidence intervals, effect sizes, degrees of freedom and *P* value noted<br>*Give P values as exact values whenever suitable.*
☒	☐	For Bayesian analysis, information on the choice of priors and Markov chain Monte Carlo settings
☐	☒	For hierarchical and complex designs, identification of the appropriate level for tests and full reporting of outcomes
☒	☐	Estimates of effect sizes (e.g. Cohen's *d*, Pearson's *r*), indicating how they were calculated

Our web collection on statistics for biologists contains articles on many of the points above.

Software and code

Policy information about availability of computer code

Data collection	No software was used in the collection of bumblebee colony performance metrics.<br><br>For the detection of pesticide residues in pollen Analyst 1.6.2 software was used to control LC– MS/MS system and for data acquisition. Quantitative and qualitative analysis was done with MultiQuant software version 3.0 . Mass Hunter software version B.07.01 to control GC-MS/MS system and for data acquisition. Quantitative and qualitative analysis was done also with Mass Hunter software. For more information, see Kiljanek et al. 2021, Talanta (https://doi.org/10.1016/j.talanta.2021.122721)<br><br>GIS were made with ArcGIS Pro 2.4.1, ESRI.
Data analysis	All data were analysed in R (version 4.1.1.) using the following packages:<br>> linear mixed effects models, 'lme4' (Bates D, Mächler M, Bolker B 2015; v. 1.1.27.1)<br>> general linear mixed effects models, 'glmmTMB' (Brooks et al. 2017; v. 1.1.3)<br>> estimated marginal means, 'emmeans' (Lenth 2022; v. 1.7.2)<br>> interaction slopes, 'interactions' (Long 2019; v. 1.1.0)<br>> model diagnostics, 'performance' (Lüdecke et al. 2021; v. 0.10.4) and 'DHARMA' (Hartig 2021; v. 0.4.5)

For manuscripts utilizing custom algorithms or software that are central to the research but not yet described in published literature, software must be made available to editors and reviewers. We strongly encourage code deposition in a community repository (e.g. GitHub). See the Nature Portfolio guidelines for submitting code & software for further information.

Data

Policy information about availability of data

All manuscripts must include a data availability statement. This statement should provide the following information, where applicable:

- Accession codes, unique identifiers, or web links for publicly available datasets
- A description of any restrictions on data availability
- For clinical datasets or third party data, please ensure that the statement adheres to our policy

The datasets analysed for the current study are available via the PoshBee project (Deliverable D1.6 Database of field records) and through FigShare: 10.6084/m9.figshare.24235573. Individual toxic endpoints (LD50s) for pesticide active ingredients came from the Pesticide Properties Database (http://sitem.herts.ac.uk/aeru/ppdb/)

Human research participants

Policy information about studies involving human research participants and Sex and Gender in Research.

Reporting on sex and gender	No human subjects were used in this research
Population characteristics	No human subjects were used in this research
Recruitment	No human subjects were used in this research
Ethics oversight	No human subjects were used in this research

Note that full information on the approval of the study protocol must also be provided in the manuscript.

Field-specific reporting

Please select the one below that is the best fit for your research. If you are not sure, read the appropriate sections before making your selection.

☐ Life sciences ☐ Behavioural & social sciences ☒ Ecological, evolutionary & environmental sciences

For a reference copy of the document with all sections, see nature.com/documents/nr-reporting-summary-flat.pdf

Life sciences study design

All studies must disclose on these points even when the disclosure is negative.

Sample size	*Describe how sample size was determined, detailing any statistical methods used to predetermine sample size OR if no sample-size calculation was performed, describe how sample sizes were chosen and provide a rationale for why these sample sizes are sufficient.*
Data exclusions	*Describe any data exclusions. If no data were excluded from the analyses, state so OR if data were excluded, describe the exclusions and the rationale behind them, indicating whether exclusion criteria were pre-established.*
Replication	*Describe the measures taken to verify the reproducibility of the experimental findings. If all attempts at replication were successful, confirm this OR if there are any findings that were not replicated or cannot be reproduced, note this and describe why.*
Randomization	*Describe how samples/organisms/participants were allocated into experimental groups. If allocation was not random, describe how covariates were controlled OR if this is not relevant to your study, explain why.*
Blinding	*Describe whether the investigators were blinded to group allocation during data collection and/or analysis. If blinding was not possible, describe why OR explain why blinding was not relevant to your study.*

Behavioural & social sciences study design

All studies must disclose on these points even when the disclosure is negative.

Study description	*Briefly describe the study type including whether data are quantitative, qualitative, or mixed-methods (e.g. qualitative cross-sectional, quantitative experimental, mixed-methods case study).*
Research sample	*State the research sample (e.g. Harvard university undergraduates, villagers in rural India) and provide relevant demographic*

Research sample	*information (e.g. age, sex) and indicate whether the sample is representative. Provide a rationale for the study sample chosen. For studies involving existing datasets, please describe the dataset and source.*
Sampling strategy	*Describe the sampling procedure (e.g. random, snowball, stratified, convenience). Describe the statistical methods that were used to predetermine sample size OR if no sample-size calculation was performed, describe how sample sizes were chosen and provide a rationale for why these sample sizes are sufficient. For qualitative data, please indicate whether data saturation was considered, and what criteria were used to decide that no further sampling was needed.*
Data collection	*Provide details about the data collection procedure, including the instruments or devices used to record the data (e.g. pen and paper, computer, eye tracker, video or audio equipment) whether anyone was present besides the participant(s) and the researcher, and whether the researcher was blind to experimental condition and/or the study hypothesis during data collection.*
Timing	*Indicate the start and stop dates of data collection. If there is a gap between collection periods, state the dates for each sample cohort.*
Data exclusions	*If no data were excluded from the analyses, state so OR if data were excluded, provide the exact number of exclusions and the rationale behind them, indicating whether exclusion criteria were pre-established.*
Non-participation	*State how many participants dropped out/declined participation and the reason(s) given OR provide response rate OR state that no participants dropped out/declined participation.*
Randomization	*If participants were not allocated into experimental groups, state so OR describe how participants were allocated to groups, and if allocation was not random, describe how covariates were controlled.*

Ecological, evolutionary & environmental sciences study design

All studies must disclose on these points even when the disclosure is negative.

Study description	In each of eight European countries (Estonia, Germany, Ireland, Italy, Spain, Sweden, Switzerland, United Kingdom) we selected 16 sites split evenly between two focal crops (Apple: N = 64, Oilseed: N = 64) following a predefined PoshBee protocol (Schweiger et al. 2019). All sites were > 3 km apart to ensure spatial independence of bumblebee foraging ranges. Sites were selected to represent a gradient of agrochemical use and proportion of cropland. in the surrounding landscape The proportion of cropland coverage ranged from 3-98% within 1-km radius buffers. See Hodge et al. (2022) for setup of the study system using a multi-actor approach.<br><br>Schweiger, O., Hodge, S., Rundlöf, M., Dominik, C. (2019) WP1.1.1 Field site selection. PoshBee protocol.<br><br>Hodge, S., Schweiger, O., Klein, A. M., Potts, S. G., Costa, C., Albrecht, M., ... & Stout, J. C. (2022). Design and planning of a transdisciplinary investigation into farmland pollinators: rationale, co-design, and lessons learned. Sustainability, 14(17), 10549.
Research sample	At each site we placed three colonies (N = 384) of Bombus terrestris terrestris, except in Ireland and the United Kingdom where the local subspecies Bombus terrestris audax was used. Colonies were all sourced through local providers. Before deployment colonies were checked for a natal queen and weighed (648 ± 70.9 g, mean ± SD). Colonies were deployed prior to crop bloom. Colonies were housed in protective structures. From each of these colonies we collected 1) metrics of colony performance and 2) pollen samples for pesticide residue analysis and palynological identification.<br><br>For metrics of colony performance we determined the weight change and maximum weight achieved by individual colonies and colony total production. For weight, we measured each colony before, during, and after bloom and took the maximum value. For weight change, we calculated the natural-log response ratio for each colony as $\ln(g\_max/g\_initial)$, where g_initial is weight prior to bloom and g_max is the maximum weight achieved by a colony during its field placement. For colony production, we closed colonies after bloom and froze them (-20 C) for laboratory dissection. From dissected colonies we counted the number of intact and eclosed worker/male and queen cocoons, including the eclosed cocoons used for nectar and pollen storage. We sum these colony structures because individual counts may be unrepresentative due to absent colony members at the time of retrieval.<br><br>After dissection, we extracted pollen stored in the colonies and pooled pollen evenly from all three colonies, aiming to obtain at least 15.0 g. Pollen samples were stored below -20 C. Samples were sent on dry ice to PIWet, where each site sample was homogenized and split for the analysis of pollen identity (1/5 sample amount) and pesticides residues (remaining sample amount). Analyses of residues were performed for samples in which quantity was at least 0.52 g (see Data Exclusions).<br><br>For pesticide residue analysis, we used a previously described method (Kiljanek et al., 2021) that is validated according to SANTE/12682/2019 (European Commission et al., 2020) and accredited in accordance with the ISO 17025 standard. Reagent blanks and blank samples were analyzed in each batch. Recovery checks with samples spiked with pesticides at LOQ levels were performed in each analytical batch to meet SANTE/12682/2019 criteria.<br><br>For pollen identification, palynological analyses were performed at the Research Centre for Agriculture and Environment (CREA) Bologna, Italy, a laboratory specialized in analyses of bees and bee products and accredited according to UNI CEI EN ISO/IEC 17025. Recognition of pollen type was based on comparison between the observed pollen forms and those present in the CREA collection of reference slides (developed using anthers of identified plant species). For each pollen type, the percentage with respect to the total number of counted pollen grains was calculated.<br><br>Land use data is based on high resolution images provided by World Imagery (ESRI) land cover features were classified at a consistent scale of 1:2500. World Imagery provides one meter satellite and aerial imagery, typically within 3-5 years of currency, using a combination of imagery sources such as 2.5m SPOT imagery and 0.5m resolution imagery from DigitalGlobe.

While performance metrics are replicated at the colony level, pesticide, pollen and landscape data are replicated at the site level. To accommodate this hierarchical data structure we used mixed effects models with site nested within country as a random factor

References:
Kiljanek, T., Niewiadowska, A., Małysiak, M., & Posyniak, A. (2021). Miniaturized multiresidue method for determination of 267 pesticides, their metabolites and polychlorinated biphenyls in low mass beebread samples by liquid and gas chromatography coupled with tandem mass spectrometry. Talanta, 235, 122721.

European Commission. Analytical Quality Control and Method Validation Procedures for Pesticide Residues Analysis in Food and Feed. (2020) SANTE/12682/2019

Sampling strategy

For site and colony sampling, we based our replication on previous power analyses performed by co-authors (EFSA 2022; Woodcock et al. 2016).

References:
European Food Safety Authority (EFSA), Auteri, D., Arce, A., Ingels, B., Marchesi, M., Neri, F.M., Rundlöf, M. and Wassenberg, J., 2022. Analysis of the evidence to support the definition of Specific Protection Goals for bumble bees and solitary bees (Vol. 19, No. 1, p. 7125E).

Woodcock, B. A., Heard, M. S., Jitlal, M. S., Rundlöf, M., Bullock, J. M., Shore, R. F., & Pywell, R. F. (2016). Replication, effect sizes and identifying the biological impacts of pesticides on bees under field conditions. Journal of Applied Ecology, 1358-1362.

Data collection

For colony performance, data was collected both in the field and in the lab by PosheBee project members and associated technicians following a predefined protocol (Rundlöf & Hodge 2019).

For pesticide residues, analyses were performed at the Department of Pharmacology and Toxicology, National Veterinary Research Institute, Puławy, which is the National Reference Laboratory for pesticide residue analysis and regularly participates in international proficiency tests with satisfactory results. Data on pesticide properties and toxicity information (used in the calculation of pesticide risk) were extracted from the Pesticide Properties Database (PPDB) hosted by the University of Hertfordshire.

For land use, all landscape features were identified and mapped within a 1-km radius, centred at the location of the sentinel bee colonies, using heads-up digitizing in a geographic information system (ArcGIS Pro 2.4.1, ESRI) and EUNIS habitat classification as a guideline. Data collection was performed by the PoshBee WorkPackage 1-2 teams.

All data collection adhered to a pre-formulated data management plan for the PoshBee project.

Rundlöf, M., Hodge, S. (2019) WP1.5.9 Assessment of Bombus terrestris colony performance and natural enemies. PoshBee protocol.

Timing and spatial scale

Colonies were deployed between 2019-03-24 and 2019-05-12 depending on region and crop specific bloom times. Similarly, colonies were retrieved between 2019-05-06 and 2019-06-12. Colonies were weighed three times i) before installment at the field sites, ii) during the middle of flowering, iii) when the colonies are retrieved from the field sites. Total production, pesticide residue and pollen identity data collection happened after colonies were retrieved from the field.

The spatial scale of the study encompasses eight European countries (see map figure in manuscript). We analyzed 106 circular landscapes with 1km radius, thus a total of 332.84 km^2 were considered.

Data exclusions

Of the 384 colonies initially deployed across 128 sites (64 apple, 64 oilseed rape), we analysed 316 colonies from 106 sites. This reduced sample size is due to colony losses (e.g., raccoon attack, overrun by machinery) or colonies not yielding enough pollen store material for pesticide quantification (N = 5 for loss, N = 63 for insufficient pollen for pesticide analyses). See Extended Data Table 3

Reproducibility

No attempts were made to repeat the full study but it was to some extent built into the experimental design by including eight countries.

Randomization

No randomization was used.

Blinding

No explicit blinding was used. However, field data collectors were not aware of the pesticide use in the focal field or the surrounding landscape, or in the pollen stores, since this data was collected through laboratory analysis after the field part of the study was completed. Conversely, laboratory data collectors were generally not aware of the colony performance measure when the pesticide residue data and pollen identity data were collected.

Did the study involve field work? ☒ Yes ☐ No

Field work, collection and transport

Field conditions

Agricultural landscapes. We chose oilseed rape and apple as our focal crops to reflect annual and perennial cropping practices and, therefore, different pest pressures, pest management strategies and pesticide use. The most dominant land covers were cropland (mean: 55%, range: 3-98%) and semi-natural habitat (mean: 37%, range: 0.1-93%), which where the latter was comprised of grasslands (mean: 19%, range: 0.1-76%), woodlands (mean: 18%, range: 0-62%), and wetlands (mean: 0.1%, range: 0-3%).

Location

Estonia, Germany, Ireland, Italy, Spain, Sweden, Switzerland, United Kingdom

Access & import/export | Access to farm locations was granted by property owners. Bumble bee colonies were sourced through local providers which held import permits.

Disturbance | No disturbance was caused by the study.

Reporting for specific materials, systems and methods

We require information from authors about some types of materials, experimental systems and methods used in many studies. Here, indicate whether each material, system or method listed is relevant to your study. If you are not sure if a list item applies to your research, read the appropriate section before selecting a response.

Materials & experimental systems

n/a	Involved in the study
☐	☐ Antibodies
☐	☐ Eukaryotic cell lines
☐	☐ Palaeontology and archaeology
☐	☒ Animals and other organisms
☐	☐ Clinical data
☐	☐ Dual use research of concern

Methods

n/a	Involved in the study
☒	☐ ChIP-seq
☒	☐ Flow cytometry
☒	☐ MRI-based neuroimaging

Antibodies

Antibodies used | *Describe all antibodies used in the study; as applicable, provide supplier name, catalog number, clone name, and lot number.*

Validation | *Describe the validation of each primary antibody for the species and application, noting any validation statements on the manufacturer's website, relevant citations, antibody profiles in online databases, or data provided in the manuscript.*

Eukaryotic cell lines

Policy information about cell lines and Sex and Gender in Research

Cell line source(s) | *State the source of each cell line used and the sex of all primary cell lines and cells derived from human participants or vertebrate models.*

Authentication | *Describe the authentication procedures for each cell line used OR declare that none of the cell lines used were authenticated.*

Mycoplasma contamination | *Confirm that all cell lines tested negative for mycoplasma contamination OR describe the results of the testing for mycoplasma contamination OR declare that the cell lines were not tested for mycoplasma contamination.*

Commonly misidentified lines (See ICLAC register) | *Name any commonly misidentified cell lines used in the study and provide a rationale for their use.*

Palaeontology and Archaeology

Specimen provenance | *Provide provenance information for specimens and describe permits that were obtained for the work (including the name of the issuing authority, the date of issue, and any identifying information). Permits should encompass collection and, where applicable, export.*

Specimen deposition | *Indicate where the specimens have been deposited to permit free access by other researchers.*

Dating methods | *If new dates are provided, describe how they were obtained (e.g. collection, storage, sample pretreatment and measurement), where they were obtained (i.e. lab name), the calibration program and the protocol for quality assurance OR state that no new dates are provided.*

☐ Tick this box to confirm that the raw and calibrated dates are available in the paper or in Supplementary Information.

Ethics oversight | *Identify the organization(s) that approved or provided guidance on the study protocol, OR state that no ethical approval or guidance was required and explain why not.*

Note that full information on the approval of the study protocol must also be provided in the manuscript.

Animals and other research organisms

Policy information about studies involving animals; ARRIVE guidelines recommended for reporting animal research, and Sex and Gender in Research

Laboratory animals	Bombus terrestris terrestris and Bombus terrestris audax colonies. The colonies were approximately 10 weeks old and contained one queen, approximately 100 workers and brood in all stages.
Wild animals	This study did not involve wild animals
Reporting on sex	*Indicate if findings apply to only one sex; describe whether sex was considered in study design, methods used for assigning sex. Provide data disaggregated for sex where this information has been collected in the source data as appropriate; provide overall numbers in this Reporting Summary. Please state if this information has not been collected. Report sex-based analyses where performed, justify reasons for lack of sex-based analysis.*
Field-collected samples	Colonies were housed in protective structures to limit weather and predator exposure. Before deployment, we confirmed that each colony had a natal queen and recorded its initial weight. No colony maintenance (e.g., supplemental feeding) was performed. At the end of the experiment colonies were weighed again, then sealed, transported from sites, and stored at -20 C.
Ethics oversight	No ethical approval was required because this study involved experimentally placed insect invertebrates.

Note that full information on the approval of the study protocol must also be provided in the manuscript.

Clinical data

Policy information about clinical studies

All manuscripts should comply with the ICMJE guidelines for publication of clinical research and a completed CONSORT checklist must be included with all submissions.

Clinical trial registration	*Provide the trial registration number from ClinicalTrials.gov or an equivalent agency.*
Study protocol	*Note where the full trial protocol can be accessed OR if not available, explain why.*
Data collection	*Describe the settings and locales of data collection, noting the time periods of recruitment and data collection.*
Outcomes	*Describe how you pre-defined primary and secondary outcome measures and how you assessed these measures.*

Dual use research of concern

Policy information about dual use research of concern

Hazards

Could the accidental, deliberate or reckless misuse of agents or technologies generated in the work, or the application of information presented in the manuscript, pose a threat to:

No	Yes	
☒	☐	Public health
☒	☐	National security
☒	☐	Crops and/or livestock
☒	☐	Ecosystems
☒	☐	Any other significant area

Experiments of concern

Does the work involve any of these experiments of concern:

No	Yes	
☒	☐	Demonstrate how to render a vaccine ineffective
☒	☐	Confer resistance to therapeutically useful antibiotics or antiviral agents
☒	☐	Enhance the virulence of a pathogen or render a nonpathogen virulent
☒	☐	Increase transmissibility of a pathogen
☒	☐	Alter the host range of a pathogen
☒	☐	Enable evasion of diagnostic/detection modalities
☒	☐	Enable the weaponization of a biological agent or toxin
☒	☐	Any other potentially harmful combination of experiments and agents
